# Quantitative analysis of lipofuscin in neurodegenerative diseases using serial sectioning two-photon microscopy and fluorescence lifetime imaging microscopy

**DOI:** 10.1117/1.NPh.12.3.035007

**Published:** 2025-08-13

**Authors:** Ayman A. Abdelhakeem, Shuaibin Chang, Anna Novoseltseva, Mackenzie Hyman, Ann C. Mckee, Irving J. Bigio, David A. Boas, Bertrand R. Huber, Hui Wang

**Affiliations:** a Boston University, Department of Electrical and Computer Engineering, Boston, Massachusetts, United States; b Boston University, Department of Biomedical Engineering, Boston, Massachusetts, United States; c VA Boston Healthcare System, U.S. Department of Veteran Affairs, Massachusetts, United States; d Boston University Chobanian and Avedisian School of Medicine, Boston University Alzheimer’s Disease Research Center and CTE Center, Massachusetts, United States; e Boston University Chobanian and Avedisian School of Medicine, Department of Neurology, Massachusetts, United States; f Boston University Chobanian and Avedisian School of Medicine, Department of Pathology and Laboratory Medicine, Massachusetts, United States; g VA Bedford Healthcare System, U.S. Department of Veteran Affairs, Bedford, Massachusetts, United States; h National Center for PTSD, U. S. Department of Veterans Affairs, Boston, Massachusetts, United States; i Massachusetts General Hospital, A.A. Martinos Center for Biomedical Imaging, Department of Radiology, Boston, Massachusetts, United States

**Keywords:** lipofuscin, neurodegeneration, neuropathology, two-photon microscopy, fluorescence lifetime imaging microscopy

## Abstract

Lipofuscin, a cellular pigment that accumulates with age, serves as a significant marker of aging. Recently, studies have linked lipofuscin with neurodegenerative diseases, such as Alzheimer’s disease (AD). Using an integrated serial sectioning optical coherence tomography (OCT) and two-photon microscopy (2PM) systems, we developed a method to examine the accumulation and distribution of lipofuscin in postmortem human brain samples. Lipofuscin was imaged with 2PM autofluorescence and quantitatively analyzed in specific structures revealed by OCT images. We involved samples from 15 people aged 60 to 90 years, including those with late-stage AD, chronic traumatized encephalopathy (CTE), and controls (NC). We developed a segmentation method for lipofuscin aggregates based on high-pass filtering and adaptive thresholding, achieving a Dice score of 61% using the integrated system at lower resolution when validated against high-resolution fluorescence lifetime imaging microscopy and phasor analysis. Quantitative metrics such as lipofuscin number density, area fraction, and radius were calculated, revealing distinct spatial distribution patterns across different brain regions and neurological conditions. AD cases exhibited a higher accumulation of lipofuscin in the gray matter sulcus regions compared with the controls, represented by the three metrics of density, area fraction, and size. The difference is particularly significant in number density. Furthermore, we discovered that lipofuscin forms layer structures in the cortical gray matter, which may be related to cell distribution in these regions. Further investigation of these areas revealed significant differences in CTE cases, especially in the infragranulary layer sulcus, compared with controls. In contrast to AD cases, the accumulation difference is significant in the sulcus of both the supergranular and infragranular layers compared with controls. These findings provide valuable information on the pathological role of lipofuscin in neurodegeneration.

## 1 Introduction

Lipofuscin is an intracellular pigment that accumulates within neurons and other cells throughout the body as a result of normal cellular metabolism and aging.^1^ It is often referred to as the “wear and tear” pigment due to its association with the accumulation of cellular waste products.^2^ Lipofuscin is commonly observed in neurons and is considered a marker of aging in the brain.^3^ Its presence has also been linked to neurodegenerative disorders such as Alzheimer’s disease (AD).^4^^,^^5^ Lipofuscin accumulates within the lysosomal compartment of neurons and other cells.^6^ Lysosomes are cellular organelles responsible for the degradation and recycling of cellular waste and damaged components.^7^ Over time, as the lysosomal degradation capacity declines with age, lipofuscin accumulates within these organelles, leading to its characteristic appearance as a granular material. The accumulation of lipofuscin is particularly prominent in long-lived postmitotic cells, such as neurons, which are unable to dilute or eliminate it through cell division. One notable characteristic of lipofuscin is its autofluorescence property.^8^ When excited by light, lipofuscin emits fluorescence in a broadband spectrum covering the green–yellow and orange–red spectral range,^9^ making it easily detectable under fluorescence microscopy. This autofluorescence arises from the accumulation of fluorophores within lipofuscin granules, which include pigments derived from oxidized lipids, protein aggregates, and advanced glycation end products.^10^ Using our integrated serial sectioning optical coherence tomography (OCT) and two-photon microscopy (2PM) system,^11^ where the excitation and emission of the 2PM system are finely adjusted to capture lipofuscin with very high contrast to other fluorescence sources, researchers can study its accumulation and distribution in cells and tissues more effectively. With the capability to image large blocks of the brain, this system facilitates robust statistical comparisons across various brain regions and conditions. Building on recent advances in OCT and 2PM for large-scale neuroimaging,^12^ this work further establishes lipofuscin as a spatially resolved biomarker for neurodegenerative pathologies.

## 2 Methods

### 2.1 Samples

For the colocalization study of lipofuscin, de-identified human brain tissues were obtained from 18 cases from the Boston University Alzheimer’s Disease Research Center and UNITE brain banks, including five brains with neuropathologically confirmed AD (Braak stages III to VI) without comorbidities, five age-matched brains with neuropathologically confirmed chronic traumatized encephalopathy (CTE) (McKee stages III to IV) without co-morbidities, and eight age-matched normal control (NC) subjects. For this study, five NC subjects were selected to match the sample size of the disease groups. Two NC samples were excluded due to identified encephalopathy or mild cognitive impairment, and one NC sample was excluded due to the young age <60. The conclusive dataset consisted of three male and two female subjects with late-stage AD, aged between 76 and 87 years, five male subjects with late-stage chronic traumatic encephalopathy, aged 75 to 89 years, and five male normal control brains aged 62 to 80 years. The tissues were fixed by immersion in 10 formalin for at least two months 43. In addition, the post-mortem interval did not exceed 24 h.

### 2.2 Imaging System Description

In this study, we employed an integrated serial-sectioning OCT and 2PM system,^11^ optimized for large tissue block imaging. This system combines OCT and 2PM to achieve balanced resolution and imaging depth for both modalities. 2PM is equipped with dual detection channels, effectively capturing lipofuscin autofluorescence via the long-wavelength 600±100  nm channel. It provides 2.4  μm lateral and 48  μm axial resolution. The $3\times 3\;{\mathrm{mm}}^{2}$ FOV has a 3  μm pixel size and 10% overlap between tiles, enabling comprehensive analysis of large brain tissue samples. OCT provides 5  μm resolution in both lateral and axial dimensions, with a confocal parameter measured at 150  μm. Block-face imaging and serial sectioning were performed on the human brain blocks. The workflow has been documented in prior studies.^13^^–^^15^ In this process, the brain tissue block was mounted and immersed in phosphate-buffered saline (PBS). A customized vibratome slicer^16^ with a 63.5 mm sapphire blade (DDK, Inc.) was positioned under the OCT-2PM imaging head, to facilitate cutting a slice from the top of the tissue block upon completion of each en face scan of the sample surface. The imaging depth for each OCT scan (the A-scan length) is 150  μm. Consequently, the 2PM focus is adjusted three times for imaging the corresponding 150  μm of brain tissue before slicing. Customized stage and vibratome control software was embedded within the image acquisition software for fully automated serial-sectioning acquisition. A parallelized post-processing script written in MATLAB was then executed to stitch together the individual image tiles to reconstruct the full volumetric image of the entire sample. For OCT images, we extracted the optical property of the scattering coefficient by following a previously established procedure^15^ and used the scattering coefficient to segment gray matter, white matter, and supragranular and infragranular layers in the cortex (Fig. S7 in the Supplementary Material). The OCT system plays a crucial role in structural segmentation across different brain regions, whereas its capability to image up to a $4\times 4\times 2\;{\mathrm{cm}}^{3}$ brain sample is essential for studying large brain areas, enabling meaningful statistical comparisons among different regions.

### 2.3 Identification of Lipofuscin Using Fluorescence Lifetime Imaging Microscopy

Fluorescence lifetime imaging microscopy (FLIM) and sub-micron two-photon imaging were carried out on the tissue slices collected above using a commercial Bruker Two-Photon system to validate the presence of lipofuscin. A 20× water immersion objective with a numerical aperture (NA) of 0.6 was used. The lateral resolution was 0.5  μm, and the axial resolution was 3  μm, with a pixel size of 0.26  μm, covering a field of view (FOV) of $270\times 270\;{\mu m}^{2}$. The excitation wavelength was 820 nm, and an emission filter of 500 to 550 nm was used. FLIM acquisition used the Prairie View software with a 250 KHz sampling rate capturing a single frame, and FLIM processing used the SPCImage software where we used the maximum-likelihood algorithm for the data fitting. Both Pairie and SPCImage are commercial software from Bruker Inc.

### 2.4 Image Segmentation

Segmentation of the lipofuscin image from the integrated OCT-2PM system was based on high-pass filtering of the image followed by adaptive thresholding. Specifically, the process was carried out in the following steps: (a) low-pass filtering was applied on the 2PM long-wavelength channel using the mean smoothing function of ImageJ with a kernel size of 50  μm. This empirical value was chosen to remove the low-frequency background variations while maintaining feature details with high frequency, including the lipofuscin and tissue boundary. (b) The original image on the 2PM long-wavelength channel was divided by the filtered image in (a). This way we normalize the low-frequency background. (c) The resulting image from (b) was binarized using the threshold function of ImageJ. We selected the Huang method^17^ for adaptive thresholding in ImageJ. We used the lower bound 1.15, which gave the best discrimination between lipofuscin and background validated by the FLIM image. As the background was normalized in (b), the adaptive thresholding gives excellent discrimination of lipofuscin across the image. In addition to lipofuscin segmentation, structural segmentation was performed on OCT images to leverage the scattering map for anatomical discrimination. The OCT scattering map effectively distinguished between gray matter and white matter in the brain. Within the gray matter, laminar structures were clearly visualized, enabling segmentation of both the infragranular and supragranular layers. These segmented regions were used to create masks, which facilitated the calculation of lipofuscin concentration in these distinct structures (Fig. S7 in the Supplementary Material). This approach allowed for a detailed and region-specific analysis of lipofuscin distribution, advancing our understanding of its localization in brain tissue with different diseases.

### 2.5 Lipofuscin Validation

To validate the segmentation method, we employed two approaches: (a) manual segmentation and (b) FLIM. (a) We manually segmented lipofuscin from a $3\times 3\;{\mathrm{mm}}^{2}$ FOV image and compared it to the result obtained from our automatic segmentation method. (b) We further validated our segmentation method by FLIM, combining the time-domain multiexponential decay analysis with phasor analysis. The same FOV of our custom 2-photon (2P) system was imaged once again using a commercial 2P Bruker system, with a lateral resolution of 0.5  μm, which is ∼4 times the resolution of our integrated system. We used the FLIM phasor domain image as a ground truth to capture lipofuscin. The FLIM phasor segmentation is done by circumscribing a region on the phasor plot. To optimize fitting accuracy, the ROI (typically an ellipse) is iteratively refined by adjusting its axial dimensions and centroid position. This systematic optimization targets a mean lifetime of ∼400 picoseconds while minimizing $\chi^{2}$ values, thereby ensuring highly accurate exponential decay fitting. The segmented image was registered to the segmentation of the integrated system using a nonlinear registration.^18^ The dice coefficient was calculated for the two validation methods.

### 2.6 Quantitative Lipofuscin Metrics

Using the segmented lipofuscin particles obtained from the integrated system, several quantitative metrics can be calculated to evaluate the spatial distribution of lipofuscin. These include the mean radius map of lipofuscin, the number density map, and the area fraction map. These metrics were derived using a sliding window approach with a $100\times 100\;\mu m^{2}$ region of interest (ROI). For the mean radius map, the connected components corresponding to lipofuscin particles within each ROI were identified. In addition, the equivalent radius of each particle was calculated based on its pixel area using sqrt $\text{area}\times \frac{4}{\pi }$, and the mean radius of all particles in the ROI was recorded. For the density map of numbers, the total count of lipofuscin particles in the ROI was normalized to the size of the ROI to compute the density of the particles. For the area fraction map, the total pixel area occupied by lipofuscin was divided by the ROI area and expressed as a percentage. The sliding window was applied throughout the image with a 50% overlap between adjacent windows, ensuring a detailed and continuous spatial representation.

### 2.7 Statistical Analysis

Statistical comparison of the three neurological groups was performed in the IBM SPSS statistics software. Considering the small sample size (five per case), the distribution may not be Gaussian. To consider that, we used the two-tailed Mann–Whitney test, which is more suitable for a skewed distribution.

## 3 Results

### 3.1 Using FLIM and Phasor Analysis to Identify Lipofuscin in Autofluorescence Images

**Fig. 1:**
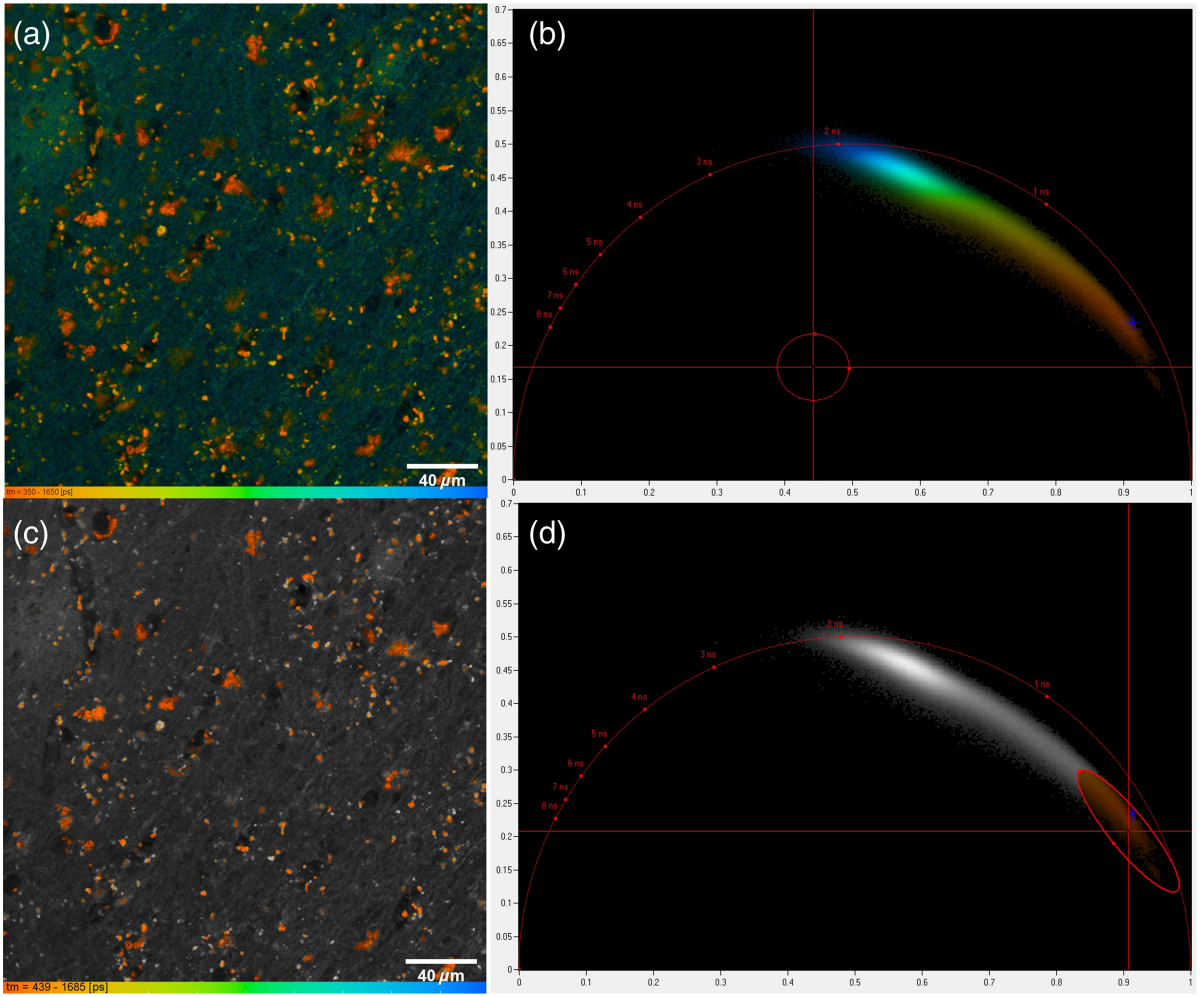
Identification of lipofuscin. (a) Fluorescence lifetime imaging (FLIM) of human brain tissue taken from a gray matter region. Lipofuscin is the orange aggregate. Elastin and Collagen appear as green to blue with a longer fluorescence lifetime. (b) Phasor plot of FLIM. Lipofuscin forms a cluster in the phasor plot. (c) Segmentation of lipofuscin based on the phasor analysis. Bright orange spots are lipofuscin. (d) Cluster segmentation of lipofuscin in the phasor domain.

**Fig. 2:**
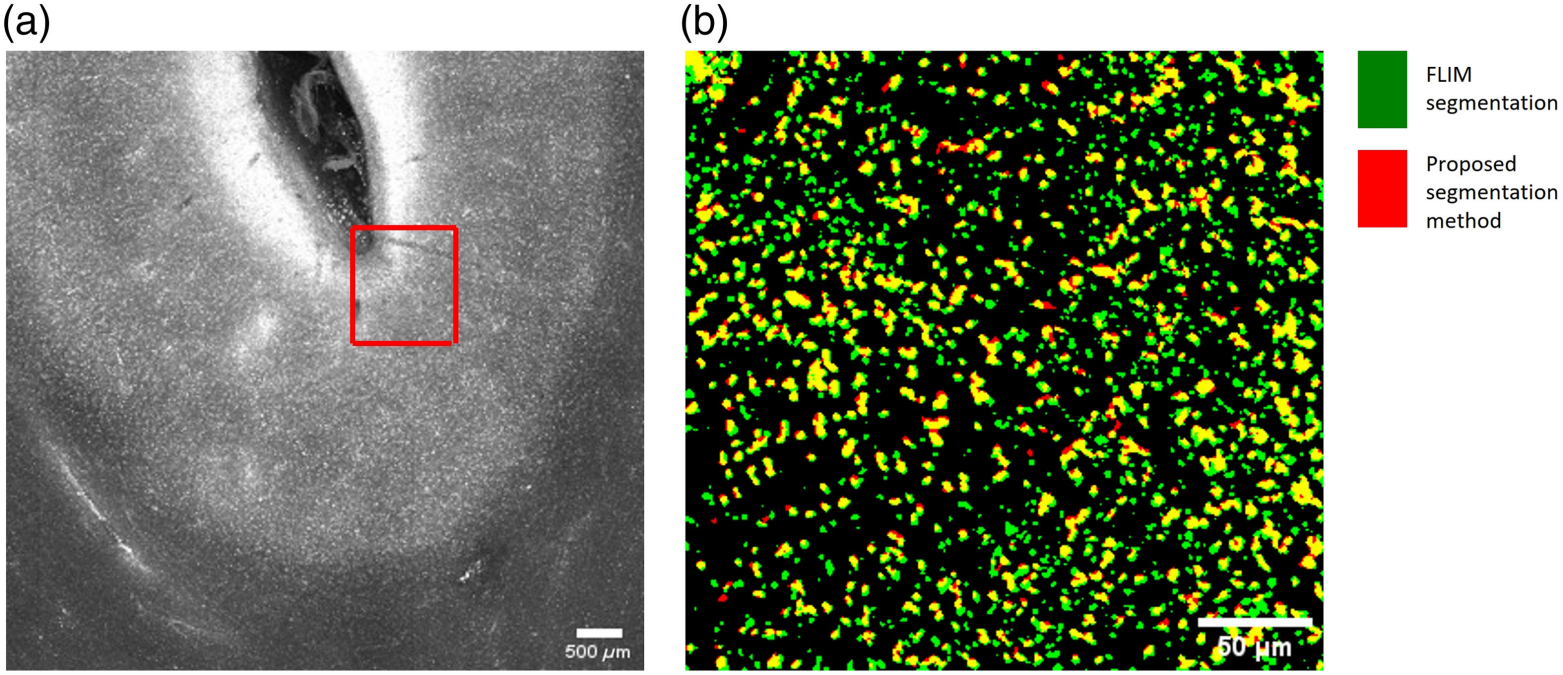
Validation with FLIM. (a) 2P auto-fluorescence image. (b) Zoom in on the red ROI at the sulcus showing the lipofuscin overlies from FLIM segmentation and developed segmentation methods.

Lipofuscin can be identified by fluorescence microscopy by its bright and broad emission spectra, which extend from 500 to 700 nm and even beyond.^1^^,^^9^ Lipofuscin appears as granular particles in brain tissue, making it distinctive from other autofluorescence structures such as tube-like elastin and collagen. Lipofuscin can also be identified using FLIM due to its short fluorescence lifetime of 400 picosecond,^9^^,^^11^ shorter than other autofluorescence molecules. We combine time-domain multiexponential decay analysis with phasor analysis to segment lipofuscin^19^ from other fluorophores such as elastin and collagen. Phasor analysis expresses the decay data in individual pixels as phase and amplitude values of a periodic waveform on a polar plot. Pixels with a similar decay signature form clusters in the phasor plot. By utilizing this technique, the distinct clustering of lipofuscin in the phasor plot allows for clear differentiation from other fluorophores. By fitting the raw image using a maximum likelihood algorithm, as shown in Fig. 1(a), we can observe that different fluorophores exhibit distinct lifetimes, with lipofuscin having a lifetime of around 400 picoseconds. Furthermore, analyzing the image in the Fourier domain [Fig. 1(b)] and using the color coding in the phasor plot provide another degree of freedom to represent the lifetime. By segmenting the lipofuscin clusters in the phasor plot [Fig. 1(d)], we see a clear delineation of the lipofuscin aggregates in the image as distinct from the background signal [Fig. 1(c)]. In particular, the background signals originate from elastin and collagen and can also be identified^20^ using the phasor analyses as illustrated in Fig. S1 in the Supplementary Material. We used the combined time domain-phasor analysis technique to validate our segmentation method for the integrated system, achieving a Dice coefficient of 61% (Fig. 2). The deviation comes mainly from a mismatch in the resolution, as the segmentation on the integrated system images missed small lipofuscin particles. Compared with manual segmentation, the dice score was lower at 52.6%, possibly attributed to the difficulty of manual annotation to capture small and dense particles.

### 3.2 Quantitative Lipofuscin Distribution in AD and CTE

**Fig. 3:**
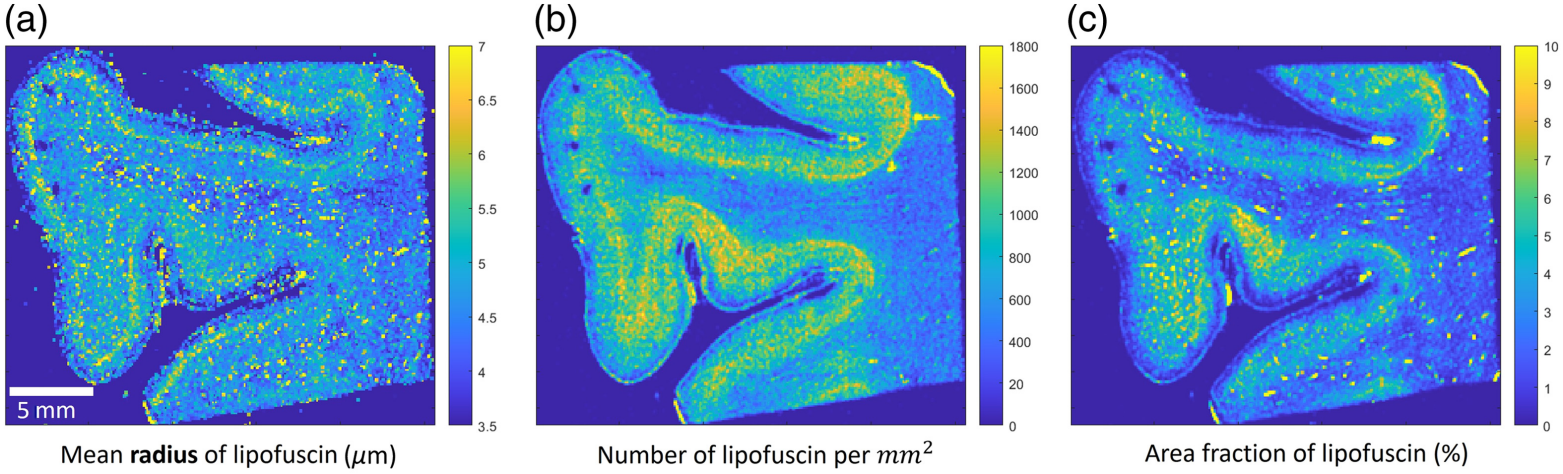
Quantitative metrics of lipofuscin. (a) Mean radius of lipofuscin (μm). (b) Number of lipofuscin $\mathrm{per}\;{\mathrm{mm}}^{2}$. (c) Area fraction of lipofuscin (%).

**Fig. 4:**
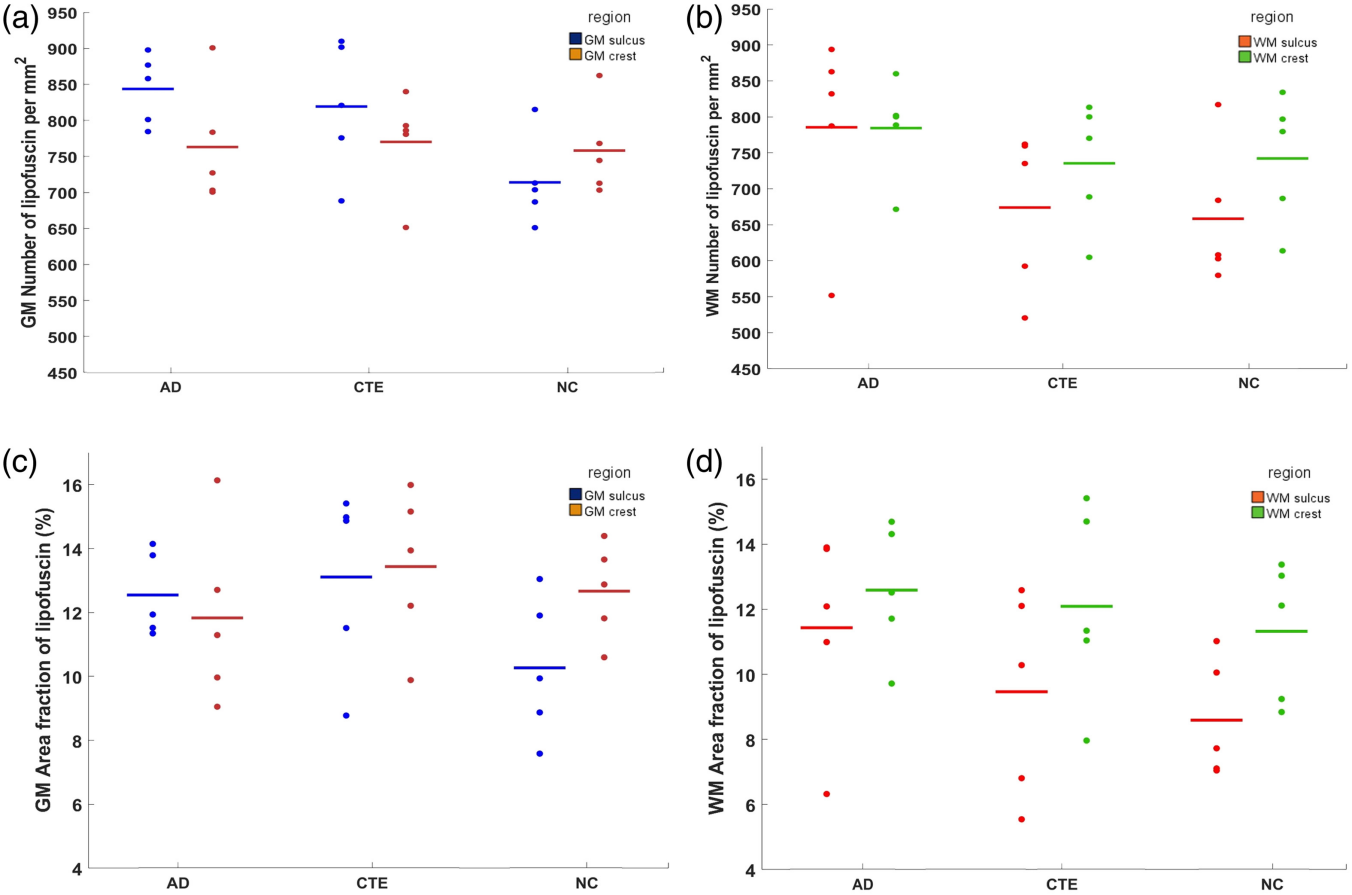
Comparison of the quantitative measurement of lipofuscin in the gray and white matter. (a), (b) Number of lipofuscins $\mathrm{per}\;{\mathrm{mm}}^{2}$ in the gray matter and white matter respectively among the AD, CTE, and NC cases. (c), (d) Area fraction (%) of lipofuscin in the gray matter and white matter, respectively, among the AD, CTE, and NC cases. A quantitative statistical comparison of the groups is provided in Table S1 in the Supplementary Material.

**Fig. 5:**
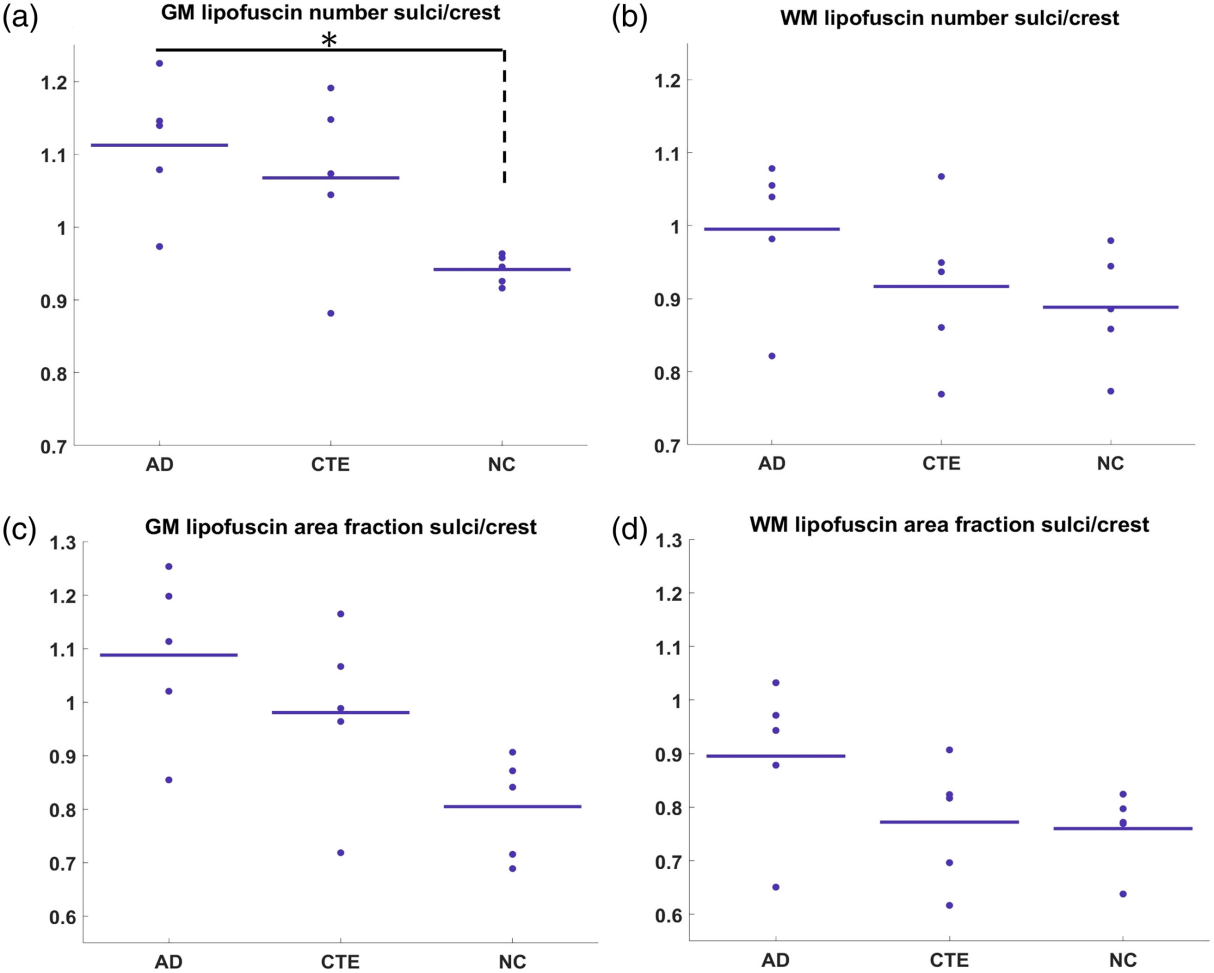
Normalized comparison for the quantitative measurement of lipofuscin. (a), (b) Comparison of the sulcus to crest ratio of the number of lipofuscin $\mathrm{per}\;{\mathrm{mm}}^{2}$ among AD, CTE, and NC in white and gray matter. (c), (d) Comparison of the sulcus to crest ratio of the lipofuscin area fraction among AD, CTE, and NC in white and gray matter. Asterisks (*) indicate significant group differences with p-value<0.05. See Table S2 in the Supplementary Material for the statistical comparison of the groups.

Figure 3 presents the three quantitative metrics calculated from an example of one of the 15 samples. From left to right are the mean radius, the number density, and the area fraction. The radius map clearly reveals a layer structure in the gray matter with a larger lipofuscin size in the middle of the gray matter. This layer is clear in all 15 samples that we imaged, suggesting that it may be related to a common structure in the cortex, possibly pyramidal neurons in layer III of the cortical cortex. The number density map highlights gray matter, suggesting the gray matter has a denser lipofuscin population compared with white matter. Within the gray matter, there are also variations of lipofuscin density, and some regions appear to have more lipofuscin. The area fraction map shows two higher density lipofuscin layers in the gray matter. These two layers may correspond to layer III with larger lipofuscin particles and infragranular layers with smaller but denser lipofuscins, respectively. It also highlights the gray matter region that has more lipofuscin density as in the number density map, with a slightly higher contrast. With these three quantitative metrics, we can compare the statistical differences among AD, CTE, and NC cases in terms of lipofuscin distribution. We manually identified regions of interest (ROIs) within the crest and sulcus areas of both gray and white matter across 15 subjects. The volume sizes ranged from 10 to 25 slices, and we calculated an average value within each ROI. Using these ROIs, we calculated the average metric in four regions of the brain, the crest of both gray matter (GM) and white matter (WM), and the sulcus of GM and WM for AD, CTE, and NC cases. By analyzing each metric, we observe distinct patterns across the brain tissue. Figure 4 presents a comparison of the number density [Figs. 4(a)–4(b)] and the area fraction [Figs. 4(c)–4(d)] among AD, CTE, and NC cases across the four regions. In the WM sulcus, the mean values for AD cases were higher than those of NC for both area fraction and number density metrics, with the area fraction showing a greater increase of 33%. In the GM sulcus, a substantial difference was observed between AD and NC for both metrics, particularly in number density (p-value of 0.06, Table S1 in the Supplementary Material). For CTE cases, the area fraction and number density in the GM sulcus were also elevated compared with NC, with increases of 27% and 15%, respectively. Notably, the increase in lipofuscin in AD cases was primarily localized to the sulcus regions of both white and gray matter, whereas no differences were detected in the crest across all metrics. The radius distribution (Fig. S2 in the Supplementary Material) showed subtle differences between disease cases and controls. Considering the unchanged crest, we can use it as a normalization factor to help control for variations in absolute lipofuscin load across subjects. Figures 5(a)–5(b) illustrate the sulcus-over-crest-ratio (SOCR) in both white and gray matter for the number density and the area fraction. This normalized quantity controls for subject variability and any potential variation in image settings between samples and produces results that depend only on pathology. We found that, in all four SOCR metrics, the value is higher for AD and CTE than NC in the GM and higher for AD in the WM compared with NC. The results agree with the above findings that AD has more lipofuscin in both GM and WM sulcus. Particularly, after normalization, the SOCR of number density shows a significant difference between AD and NC in the GM (p<0.05) (Table S2 in the Supplementary Material). By contrast, CTE has a pronounced difference shown only in the GM. The SOCR of CTE, although also being higher than NC, does not appear to have statistical significance compared with NC. The results further consolidate that lipofuscin in the two neurodegenerative diseases accumulates aggressively in the sulcus compared with the crest, and the gray matter sulcus is the region with the most vulnerable region in terms of lipofuscin accumulation. Furthermore, the radius measurements have shown the same trend with the disease but with less significance (Fig. S3 in the Supplementary Material).

### 3.3 Layer-Specific Lipofuscin Distribution in AD and CTE

**Fig. 6:**
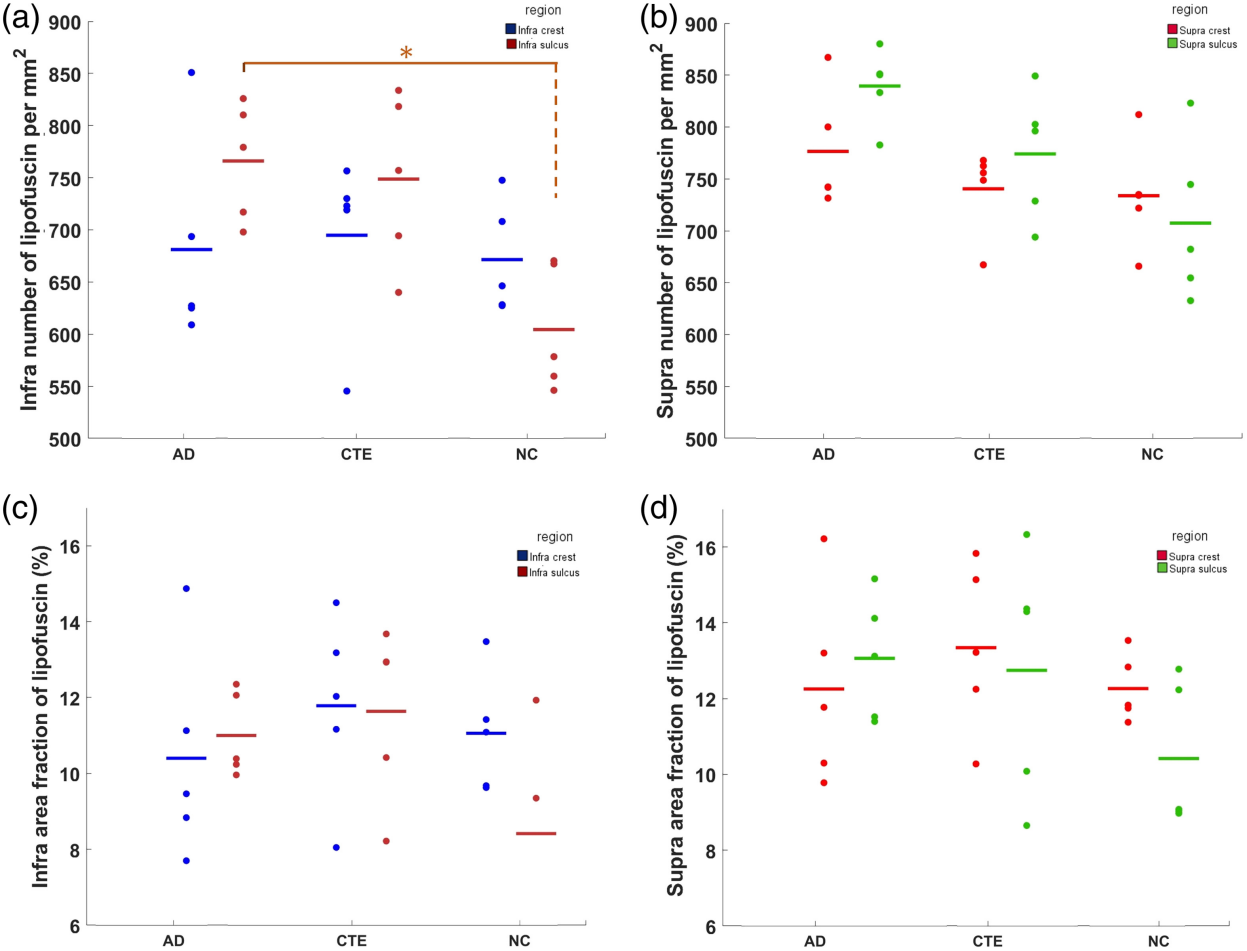
Comparison for the quantitative measurement of lipofuscin supra and infra in the gray matter. (a), (b) Number of lipofuscin $\mathrm{per}\;{\mathrm{mm}}^{2}$ of in the gray matter infra and supra respectively among the AD, CTE, and NC cases. (c), (d) Area fraction (%) of lipofuscin in the gray matter infra and supra respectively among the AD, CTE, and NC cases. Asterisks (*) indicate significant group differences with p-value<0.05.

**Fig. 7:**
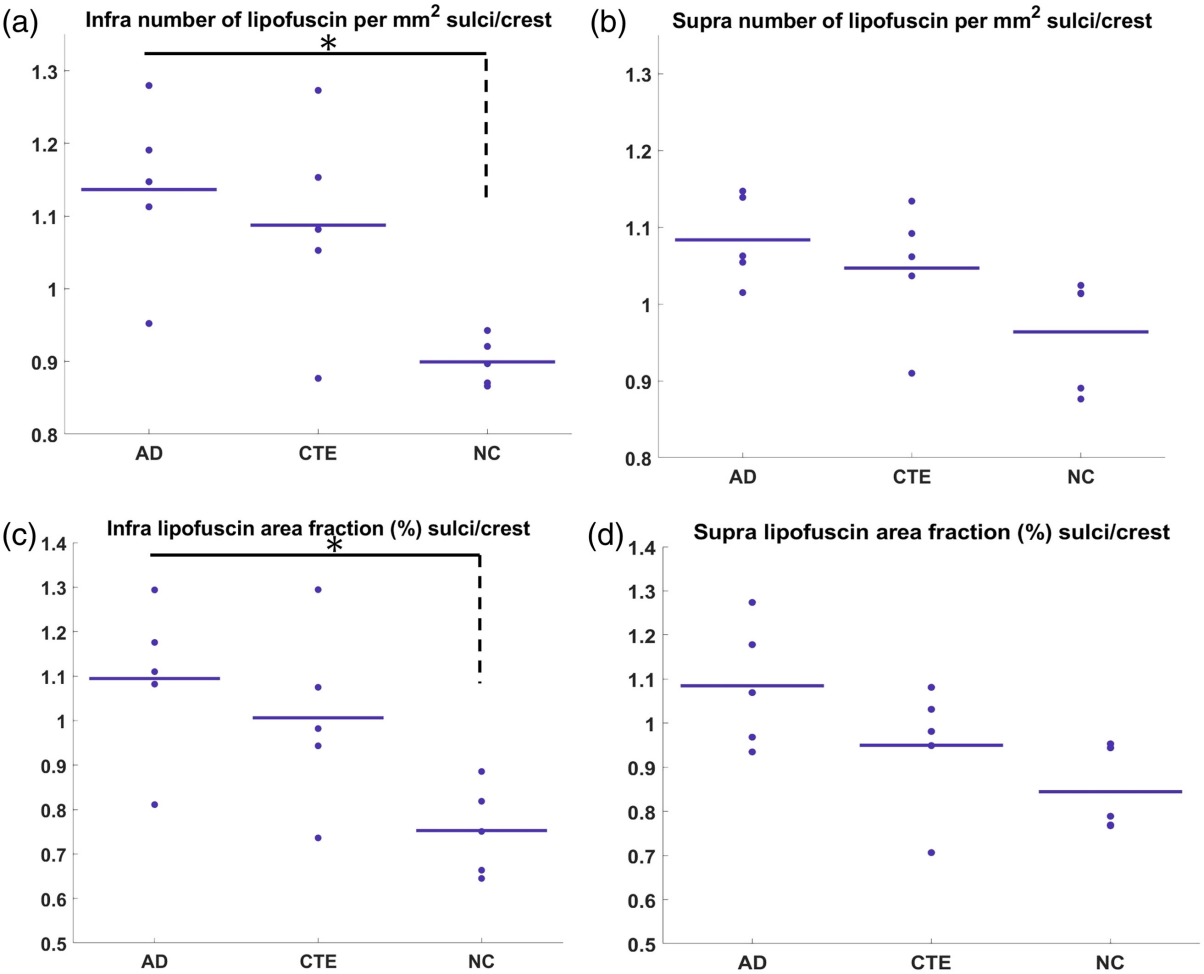
Normalized comparison for the quantitative measurement of lipofuscin in the gray matter layers. (a), (b) Statistical comparison of the sulcus to crest ratio of the number of lipofuscins $\mathrm{per}\;{\mathrm{mm}}^{2}$ among AD, CTE, and NC in infra and supra layer respectively. (c), (d) Comparison of the sulcus to crest ratio of the lipofuscin area fraction among AD, CTE, and NC in the infra and supra layer, respectively. Asterisks (*) indicate significant group differences with p-value<0.05.

The intraneuronal accumulation of lipofuscin is one of the most evident features in aged brain tissue.^21^ In the cerebral cortex, neurons form distinctive laminar structures that could result in a differentiation in a layer-specific distribution of lipofuscin.^22^^,^^23^ Particularly, the role of differed distribution in neurodegeneration^3^ is intriguing for in-depth investigation. We segmented the cortex into the supragranular and infragranular layers based on the OCT structural images. We found that the lipofuscin in the superficial layer was sparsely distributed, whereas in the deeper layers, the small lipofuscin particles were densely distributed. Figure 6 shows the comparison in the area fraction and number density for the infragranular and supragranular layers. Notably, both supragranular and infragranular layers of the sulcus showed significantly more number density (p<0.05) (Table S3 in the Supplementary Material) in AD than in NC. In the area fraction [Figs. 6(c)–6(d)], both infra and supra layers exhibited 30% higher lipofuscin levels in the suclus in AD compared with NC, although not significantly different. Notably, there were significant differences between CTE and NC in the infragranular layer of the sulcus in multiple quantitative metrics, including number density (p<0.05) and area fraction (p<0.05). Intriguingly, the radius showed a similar trend as well (p=0.05) (Fig. S4 in the Supplementary Material). By contrast, the lipofuscin distribution in the crest does not show a difference among groups. Considering the unchanged crest condition in diseases, we can use it as a normalization factor as we did before. Figures 7(a)–7(d) illustrate the SOCR metric in both the infragranular and supragranular layers in the gray matter for the number density and the area fraction; we see that the ratio is greater than one for both AD and CTE and less than one in NC mostly in both metrics of number density and area fraction. Particularly, the SOCR value presented significant differences (p<0.05) between the disease and NC in multiple metrics (Table S4 in the Supplementary Material). By contrast, the mean radius of lipofuscin was less affected than the other two metrics (Fig. S5 Supplementary Material).

### 3.4 Age-Related Lipofuscin Accumulation in AD and CTE

**Fig. 8:**
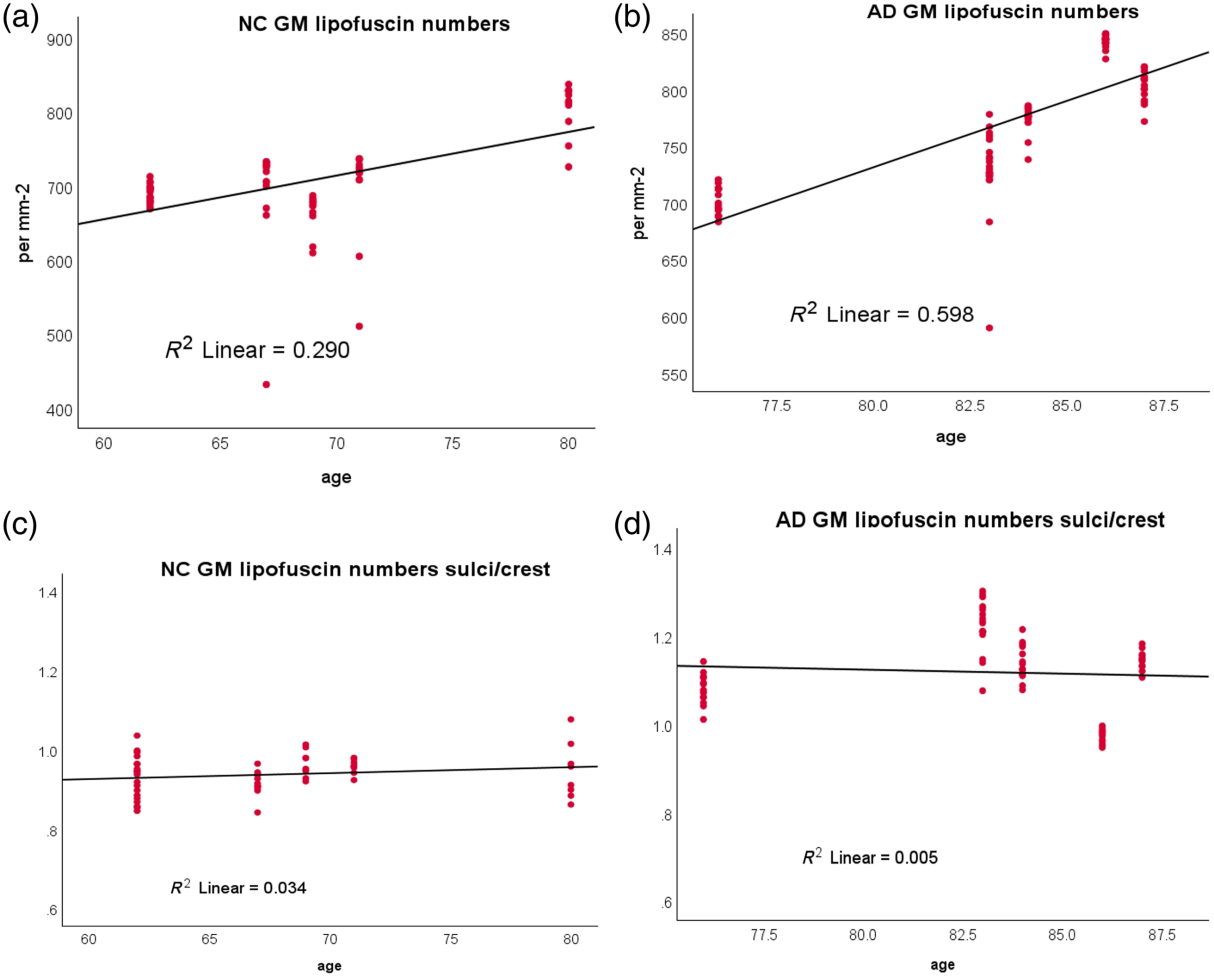
Linear regression of lipofuscin number metric with aging for gray matter. The $R^{2}$ of the least square fitting is reported for each figure for AD and NC cases.

We previously reported a trend of increasing lipofuscin with age, particularly in white matter, with a steeper slope than in gray matter.^11^ In this current study, we expand upon that prior finding and find an increasing level of lipofuscin in both gray and white matter with respect to age in AD samples (Fig. 8). The increasing rate is approximately the same between AD and NC samples. As the age distribution of AD samples is slightly higher than that of NC, we further confirmed that the increasing level of lipofuscin in AD compared with NC was not simply a result of increased age, rather localized lipofuscin accumulation is specific AD. To this end, we plotted the SOCR for all three lipofuscin metrics and examined the change in ratio over age. We discovered that, unlike the overall increase of lipofuscin associated with aging, the SOCR remained largely unaffected by age, although the SOCR value is higher and the variation is greater in AD. This result further strengthens the use of SOCR as a biomarker as lipofuscin is highly accumulated in the sulcus region of AD brains. The age effect on lipofuscin in CTE cases is much more subtle in all three calculated metrics (Fig. S6 in the Supplementary Material).

## 4 Discussion

We have demonstrated the label-free large-scale imaging of lipofuscin aggregates in human brain tissue using the two-photon autofluorescence microscope of our integrated OCT-2PM system and validated the results using FLIM and phasor analysis, as well as manual annotation. The excellent discrimination of lipofuscin from the background signal in the home-built OCT-2PM system enables adaptive threshold-based automatic segmentation. The autofluorescence signal was stronger at the tissue boundary likely due to the thinner tissue surface, resulting in increased background noise. Consequently, the threshold technique might miss some lipofuscin at the tissue boundary. The validation steps confirm the reliability and effectiveness of our segmentation method, highlighting its potential utility in studying lipofuscin accumulation and distribution. The phasor analysis on the FLIM is particularly useful because different phasor clusters can be selected, and the corresponding pixels can be back-annotated in the time-domain FLIM images. The decay functions of pixels within the selected phasor range can be combined into a single decay curve with high photon numbers ensuring sufficient signal-to-noise ratio for robust lifetime analysis (Fig. S8 in the Supplementary Material). Thus, phasor analysis is presented as a method for segmenting different fluorescence structures, offering a powerful complement to traditional time-domain analysis methods.^24^ The long-tail pattern in the phasor plot of lipofuscin is related to lipofuscin’s inherent heterogeneity in composition and structure. Lipofuscin is composed of oxidized proteins (30% to 70%), lipids such as triglycerides, free fatty acids, cholesterol, lipoproteins (20% to 50%), and a smaller amount of carbohydrates. This diverse chemical composition naturally contributes to heterogeneous fluorescence behavior.^25^^,^^26^ Furthermore, applying a multicomponent maximum likelihood algorithm for fitting and segmenting lipofuscin clusters confirms a histogram peak at 400 picoseconds, consistent with prior studies that identify this lifetime as a defining feature of lipofuscin.^11^ It is worth mentioning that several factors contributed to the observed Dice coefficients. The higher resolution of the Bruker system allowed small lipofuscin particles to be identified, which is beyond the resolution of the integrated system. In addition, FLIM was conducted on thin slice sections obtained after OCT-2PM imaging; therefore, distortion occurred during sample mounting on a glass slide before imaging again using FLIM. The registration might not co-align the 2PM autofluorescence and FLIM images for a single cell. Despite these challenges, our method demonstrated a Dice coefficient of 61% in segmenting lipofuscin when compared with the FLIM phasor segmentation. Comparison with manual segmentation yielded a lower Dice coefficient. This was due to the difficulty of visually identifying all small-sized lipofuscin granules in the image obtained from the integrated system.

The spatial distribution of lipofuscin and the differences between AD and NC have been previously reported. Mann et al.^27^ in 1984 evaluated the nucleus basalis of meynert in AD with age-matched normal control samples at ages from 50 to 90 years old and found no significant difference. However, Dowson^5^ in 1982 did find differences between AD and NC in young ages, but he also agreed that, at older ages, the difference was reduced compared with the young ages. Nevertheless, it should be noted that these studies conducted in the 1980s are limited to a small sampling size, with about only 100 neurons; consequently, the results might suffer from insufficient data. Back in the 1980s, researchers were limited by the technology available to study large volumes of brain tissue. However, our measurements cover cubic centimeters of brain tissue, providing more statistical power. Generally, our results provide more evidence that AD does have more lipofuscin accumulation than NC, but the increase in number density is more pronounced in the sulcus region of the gray matter. Meanwhile, in CTE, even fewer studies have investigated lipofuscin, which makes sense as we find there is essentially no change in lipofuscin density or area fraction in most of the regions compared with NC. With the help of OCT imaging, layer-specific segmentation enabled us to discover that the increase of lipofuscin accumulation is highly localized with layers, which only occurs in the infragranular layer of the sulcus region. Despite the small number of total brain samples, our method makes full use of the tissue block instead of small sampling areas. This advantage provides us with a large spatial coverage for studying the lipofuscin distribution and generates abundant sampling within the subject. We plan to collect more brain samples in the future.

There are several future directions we could pursue in further advancing our understanding of lipofuscin in neurodegenerative diseases. The analysis pipeline could be enhanced by utilizing recent advancements in machine learning methods that show promise for segmenting low-resolution images using prior knowledge from high-resolution images.^28^^,^^29^ Furthermore, the resolution of our 2PM on our integrated system could easily improve 2× as in the present study we under-sampled to match the acquisition speed of 2PM with OCT. For small sample blocks in which total acquisition time is less of a concern, we can improve the resolution of 2PM using a smaller voxel pitch to properly sample the optical resolution. We can also increase the optical resolution with a higher NA objective, which will help with imaging small lipofuscin particles.

## 5 Conclusion

The novelty of our methodology is the use of a combined 2PM and an OCT system to study the quantitative distribution of lipofuscin in neurodegenerative diseases. 2PM enables quantification of lipofuscin autofluorescence, whereas OCT enables structural differentiation in the dorsolateral prefrontal cortex. The combined system allows co-registered 2PM and OCT images taken at the same time and in a volumetric fashion, such that structure-specific analysis of lipofuscin distribution is performed. Our results elaborated on the importance of this structure-specific analysis as lipofuscin aggregation shows heterogeneous patterns that are more localized in certain structures than others. Based on the results of the study, we can draw several conclusions. First, autofluorescence imaging of lipofuscin resolves differences in lipofuscin accumulation in neurodegenerative diseases. Second, the AD brain shows higher levels of lipofuscin mostly in the sulcal region of the brain compared with NC brains, suggesting a correlation between AD pathology and increased lipofuscin formation. Last, CTE pathology primarily affects lipofuscin formation in highly localized brain structures, notably in the infragranular layer of the sulcus.

## Supplementary Material

10.1117/1.NPh.12.3.035007.s01

## Data Availability

All data included in this study are presented in the figures. Raw image data of the human brain may be obtained from the corresponding author following the Boston University guidelines.

## References

[1] Moreno-García A. et al., “An overview of the role of lipofuscin in age-related neurodegeneration,” Front. Neurosci. 12, 464 (2018).1662-453X 10.3389/fnins.2018.00464 30026686 PMC6041410

[2] Gray D. A. Woulfe J., “Lipofuscin and aging: a matter of toxic waste,” Sci. Aging Knowl. Environ. 2005(5), re1 (2005). 10.1126/sageke.2005.5.re1 15689603

[3] Brunk U. T. Terman A., “The mitochondrial-lysosomal axis theory of aging: accumulation of damaged mitochondria as a result of imperfect autophagocytosis,” Eur. J. Biochem. 269(8), 1996–2002 (2002). EJBCAI 0014-2956 10.1046/j.1432-1033.2002.02869.x 11985575

[4] Brückner G. et al., “Cortical areas abundant in extracellular matrix chondroitin sulphate proteoglycans are less affected by cytoskeletal changes in Alzheimer’s disease,” Neuroscience 92(3), 791–805 (1999). 10.1016/S0306-4522(99)00071-8 10426522

[5] Dowson J. H., “Neuronal lipofuscin accumulation in ageing and Alzheimer dementia: a pathogenic mechanism?” Br. J. Psychiatr. 140(2), 142–148 (1982). 10.1192/bjp.140.2.142 7074296

[6] Adler L. IV et al., “The 11-cis retinal origins of lipofuscin in the retina,” Prog. Mol. Biol. Transl. Sci. 134, e1–e12 (2015). 10.1016/bs.pmbts.2015.07.022 26310175

[7] Settembre C. et al., “Signals from the lysosome: a control centre for cellular clearance and energy metabolism,” Nat. Rev. Mol. Cell Biol. 14(5), 283–296 (2013). NRMCBP 1471-0072 10.1038/nrm3565 23609508 PMC4387238

[8] Katz M. L. Gerald Robison W. Jr., “What is lipofuscin? Defining characteristics and differentiation from other autofluorescent lysosomal storage bodies,” Arch. Gerontol. Geriatr. 34(3), 169–184 (2002). 10.1016/S0167-4943(02)00005-5 14764321

[9] Chen C. et al., “In vivo near-infrared two-photon imaging of amyloid plaques in deep brain of Alzheimer’s disease mouse model,” ACS Chem. Neurosci. 9(12), 3128–3136 (2018). 10.1021/acschemneuro.8b00306 30067906

[10] Jung T. Bader N. Grune T., “Lipofuscin: formation, distribution, and metabolic consequences,” Ann. N. Y. Acad. Sci. 1119(1), 97–111 (2007). ANYAA9 0077-8923 10.1196/annals.1404.008 18056959

[11] Chang S. et al., “Multi-scale label-free human brain imaging with integrated serial sectioning polarization sensitive optical coherence tomography and two-photon microscopy,” Adv. Sci. 10(35), 2303381 (2023). 10.1002/advs.202303381 PMC10724383 37882348

[12] Chang S., “Serial sectioning PSOCT and 2PM for imaging post-mortem human brain and neurodegeneration,” PhD thesis, Boston University (2023).

[13] Wang H. et al., “as-PSOCT: volumetric microscopic imaging of human brain architecture and connectivity,” NeuroImage 165, 56–68 (2018). NEIMEF 1053-8119 10.1016/j.neuroimage.2017.10.012 29017866 PMC5732037

[r14] Yang J. et al., “Volumetric characterization of microvasculature in ex vivo human brain samples by serial sectioning optical coherence tomography,” IEEE Trans. Biomed. Eng. 69(12), 3645–3656 (2022). IEBEAX 0018-9294 10.1109/TBME.2022.3175072 35560084 PMC9888394

[15] Yang J. et al., “Improving the characterization of ex vivo human brain optical properties using high numerical aperture optical coherence tomography by spatially constraining the confocal parameters,” Neurophotonics 7(4), 045005 (2020). 10.1117/1.NPh.7.4.045005 33094126 PMC7575831

[16] Wang J. Li C. Chen S.-C., “Sectioning soft materials with an oscillating blade,” Precis. Eng. 56, 96–100 (2019). PREGDL 0141-6359 10.1016/j.precisioneng.2018.11.002

[17] Huang L.-K. Wang M.-J. J., “Image thresholding by minimizing the measures of fuzziness,” Pattern Recognit. 28(1), 41–51 (1995). 10.1016/0031-3203(94)E0043-K

[18] Arganda-Carreras I. et al., “Consistent and elastic registration of histological sections using vector-spline regularization,” Lect. Notes Comput. Sci. 4241, 85–95 (2006). LNCSD9 0302-9743 10.1007/11889762_8

[19] Monteleone A. et al., “Label-free identification and differentiation of different microplastics using phasor analysis of fluorescence lifetime imaging microscopy (FLIM)-generated data,” Chem. Biol. Interact. 342, 109466 (2021). 10.1016/j.cbi.2021.109466 33865829

[20] Shirshin E. A. et al., “Two-photon autofluorescence lifetime imaging of human skin papillary dermis in vivo: assessment of blood capillaries and structural proteins localization,” Sci. Rep. 7(1), 1171 (2017). SRCEC3 2045-2322 10.1038/s41598-017-01238-w 28446767 PMC5430894

[21] Koenig K. et al., “Clinical optical coherence tomography combined with multiphoton tomography of patients with skin diseases,” J. Biophotonics 2(6–7), 389–397 (2009). 10.1002/jbio.200910013 19598177

[22] D’Andrea M. R. et al., “Lipofuscin and aβ42 exhibit distinct distribution patterns in normal and Alzheimer’s disease brains,” Neurosci. Lett. 323(1), 45–49 (2002). NELED5 0304-3940 10.1016/s0304-3940(01)02444-2 11911987

[23] Brizzee K. R. Johnson F. A., “Depth distribution of lipofuscin pigment in cerebral cortex of albino rat,” Acta Neuropathol. 16, 205–219 (1970). ANPTAL 1432-0533 10.1007/BF00687360 5478209

[24] Becker W., The BH TCSPC Handbook, 10th ed. (2023).

[25] Terman A. Brunk U. T., “Lipofuscin,” Int. J. Biochem. Cell Biol. 36(8), 1400–1404 (2004). IJBBFU 1357-2725 10.1016/j.biocel.2003.08.009 15147719

[26] Scipioni L. et al., “Phasor analysis of local ICS detects heterogeneity in size and number of intracellular vesicles,” Biophys. J. 111(3), 619–629 (2016). BIOJAU 0006-3495 10.1016/j.bpj.2016.06.029 27508445 PMC4982927

[27] Mann D. M. A. Yates P. O. Marcyniuk B., “Changes in nerve cells of the nucleus basalis of Meynert in Alzheimer’s disease and their relationship to ageing and to the accumulation of lipofuscin pigment,” Mech. Ageing Dev. 25(1–2), 189–204 (1984). 10.1016/0047-6374(84)90140-4 6202988

[28] Li X. et al., “Unsupervised content-preserving transformation for optical microscopy,” Light: Sci. Appl. 10(1), 44 (2021). 10.1038/s41377-021-00484-y 33649308 PMC7921581

[29] Eun D. et al., “Deep-learning-based image quality enhancement of compressed sensing magnetic resonance imaging of vessel wall: comparison of self-supervised and unsupervised approaches,” Sci. Rep. 10(1), 13950 (2020). SRCEC3 2045-2322 10.1038/s41598-020-69932-w 32811848 PMC7434911

