# Supplementary material for: Quantitative analysis of lipofuscin in neurodegenerative diseases using serial sectioning two-photon microscopy and fluorescence lifetime imaging microscopy
Source: Neurophotonics. 2025 Aug 13;12(3):035007. doi: 10.1117/1.NPh.12.3.035007 (PMC12348049; doi:10.1117/1.NPh.12.3.035007)
Supplement: Supplementary file 1 [file NPh_012_035007_SD001.pdf]

1 Supplemental material

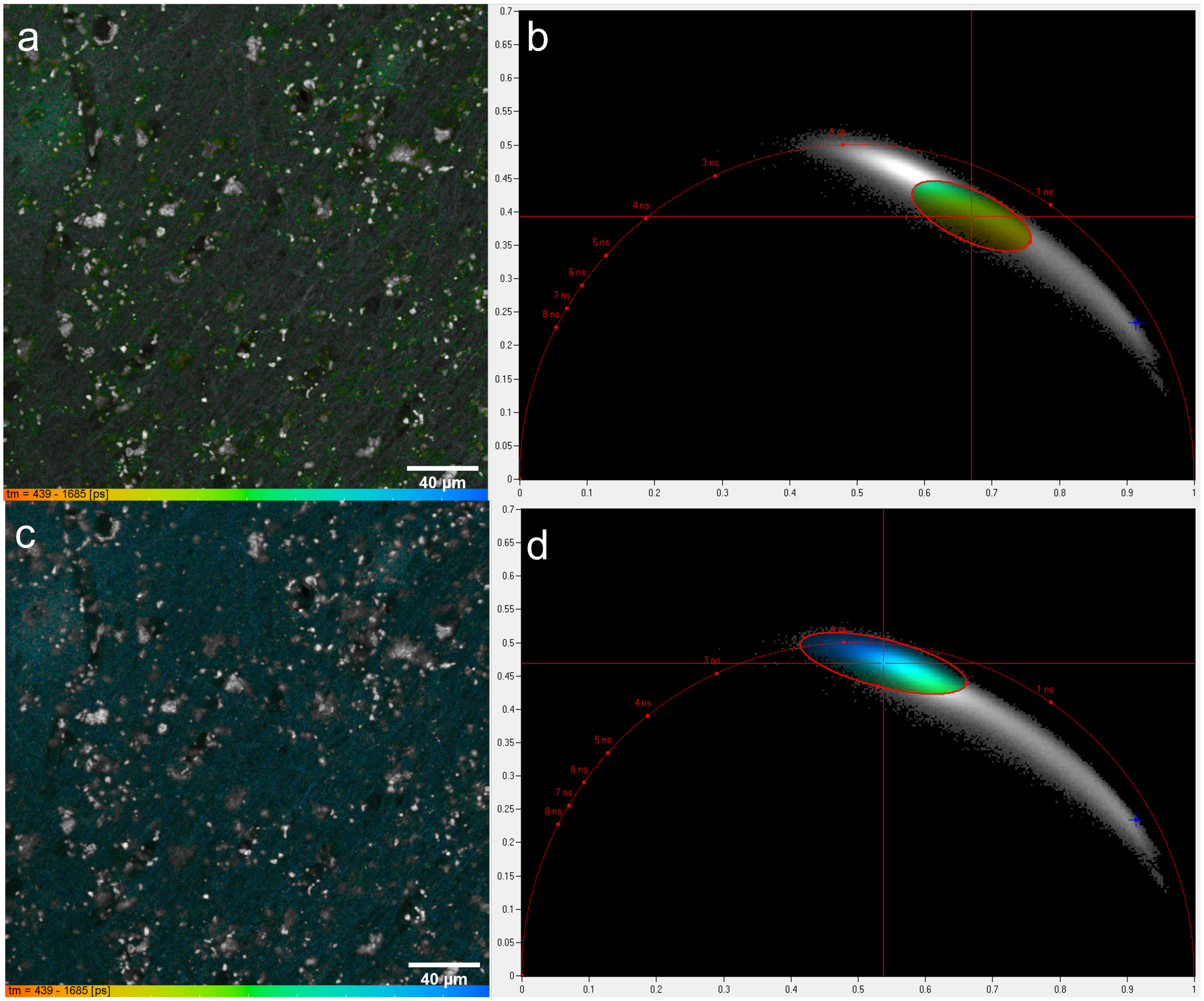

Fig 1 Identification of collagen and elastin in human brain tissue. (a) Segmentation of collagen based on the phasor analysis centered around $1080ps$. (b) Phasor plot of FLIM. collagen forming a cluster in the phasor plot. (c) Segmentation of elastin based on the phasor analysis centered around $1350ps$. (d) cluster segmentation of elastin in the phasor domain.

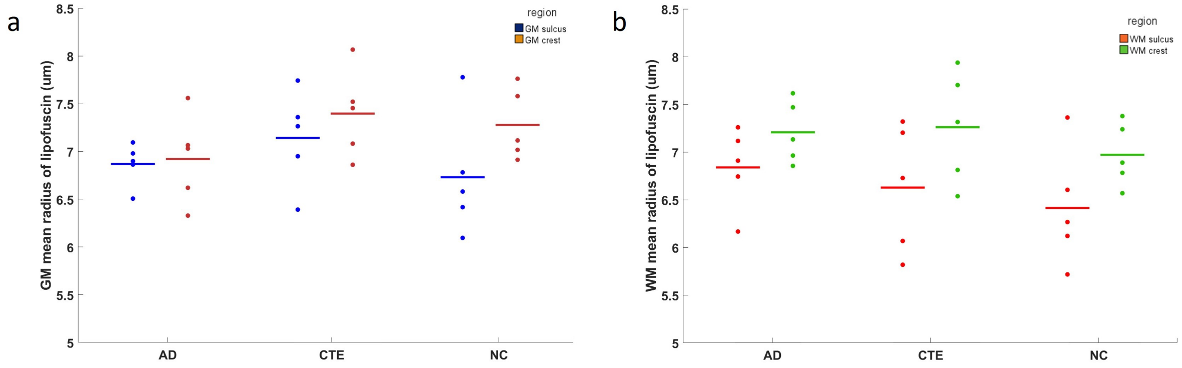

Fig 2 Comparison for the quantitative radius metric of lipofuscin in the gray and white matter: mean radius of lipofuscin per (um) in the gray (a) and white matter (b) respectively among the AD, CTE and NC cases.

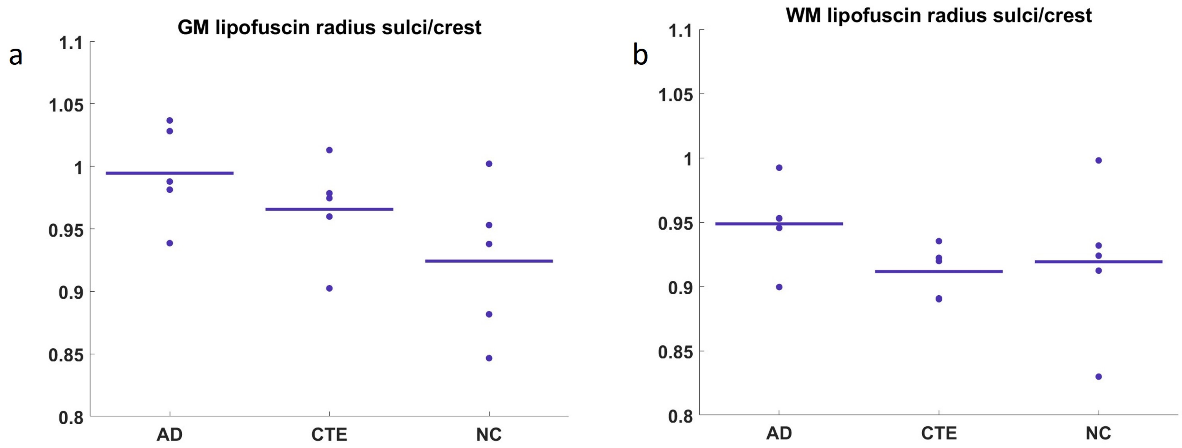

Fig 3 Normalized comparison for the quantitative radius metric of lipofuscin: the sulcus to crest ratio among AD, CTE and NC in gray (a) and white matter (b).

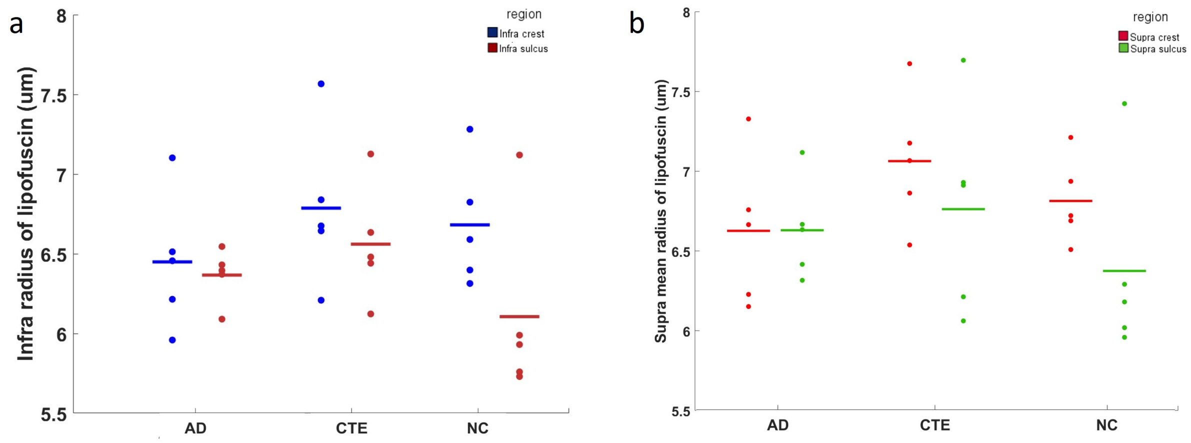

Fig 4 Comparison for the quantitative measurement of lipofuscin supra and infra in the gray matter: (a) the mean radius of lipofuscin (um) in the infragranular (a) and supragranular (b) layer of cortex respectively among the AD, CTE and NC cases.

a

AD_NC	GM_sul	GM_cre	WM_sul	WM_cre
Count	0.0624	0.9854	0.2080	0.5559
Occupation	0.3384	0.7489	0.3018	0.6528
Radius	0.7013	0.3776	0.4630	0.5086

b

CTE_NC	GM_sul	GM_cre	WM_sul	WM_cre
Count	0.1016	0.7947	0.9963	0.9963
Occupation	0.1584	0.8776	0.9130	0.8379
Radius	0.2679	0.9854	0.8776	0.5559

Table 1 P-Value for the statistical comparison of the quantitative measurement of lipofuscin in the gray and white matter between AD and NC (a) and CTE and NC (b) across 4 different regions in the brain

a

AD_NC	GM_normalized	WM_normalized
Count	0.0252	0.1584
Occupation	0.207	0.1184
Radius	0.0624	0.4192

b

CTE_NC	GM_normalized	WM_normalized
Count	0.1373	0.8776
Occupation	0.1584	0.9432
Radius	0.4630	0.7013

Table 2 P-Value for normalized statistical comparison for the quantitative measurement of lipofuscin in gray and white matter between AD and NC (a) and between CTE and NC (b).

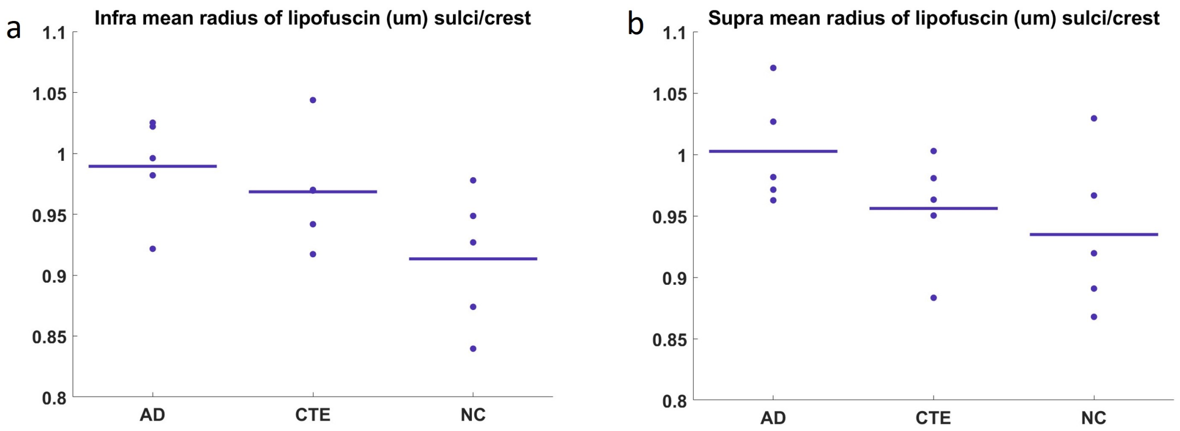

Fig 5 Normalized comparison for the quantitative measurement of lipofuscin in the cortical layers: the sulcus to crest ratio of the mean radius of lipfuscin (um) among AD, CTE and NC in infragranular (a) and supragranular (b) layer respectively.

a

AD_NC	crest_infra	crest_supra	sul_infra	sul_supra
Count	0.7947	0.3384	0.0170	0.0138
Occupation	0.7013	0.9854	0.1819	0.2080
Radius	0.6041	0.7489	0.3776	0.2679

b

CTE_NC	crest_infra	crest_supra	sul_infra	sul_supra
Count	0.7947	0.4630	0.0441	0.4192
Occupation	0.8379	0.6528	0.0305	0.2679
Radius	0.8776	0.6041	0.0526	0.3018

Table 3 P-Value for the statistical comparison of the quantitative measurement of lipofuscin in the supragranular and infragranular layer of cortex between AD and NC (a) and CTE and NC (b) across 4 different regions in the brain

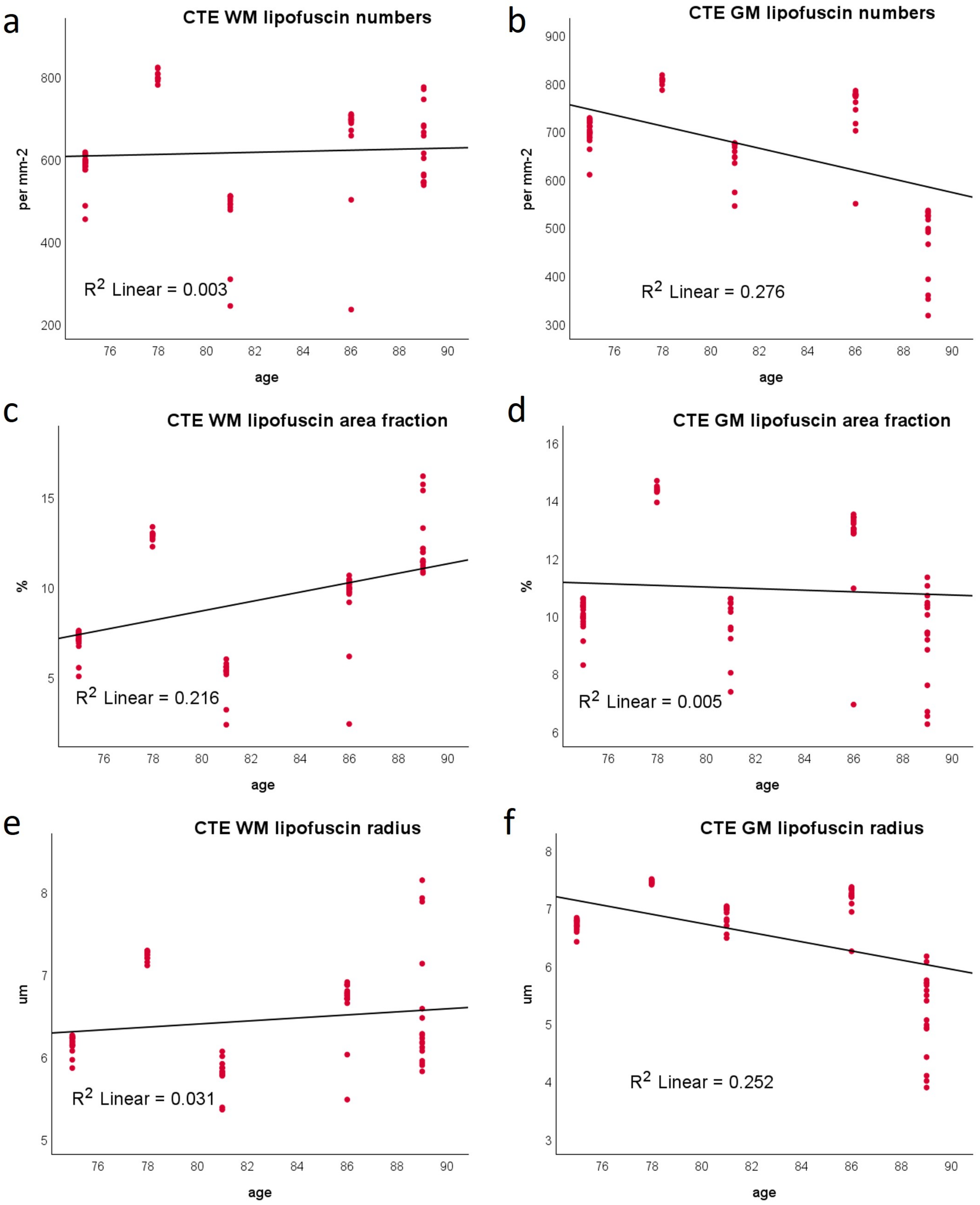

Fig 6 Linear regression of lipofuscin for the CTE disease cases shown for the three quantitative metrics lipofuscin number, area fraction, and radius with respect to age in gray matter and white matter separately. The R^2 of least square fitting is reported for each figure.

a

AD_NC	infra_normalized	supra_normalized
Count	0.0138	0.0207
Occupation	0.0252	0.0441
Radius	0.0738	0.1584

b

CTE_NC	infra_normalized	supra_normalized
Count	0.0868	0.1016
Occupation	0.1373	0.3018
Radius	0.4192	0.8776

Table 4 P-Value for normalized statistical comparison for the quantitative measurement of lipofuscin in infragranular and supragranular layer of cortex between AD and NC (a) and CTE and NC (b).

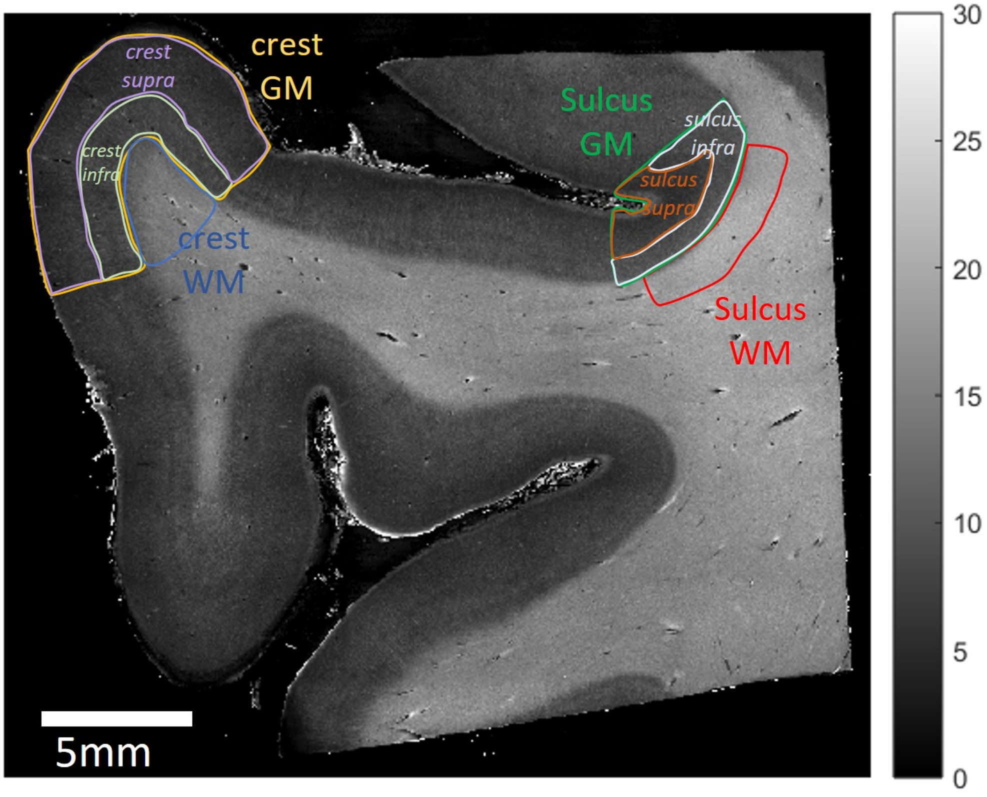

Fig 7 Optical coherence tomography (OCT)–derived scattering coefficient (μs) map of a brain cortical section with overlaid annotations demarcating the gyral crest and sulculs regions, which are each segmented into gray matter (GM) and white matter (WM) and further subdivided into supra- and infra- subregions.

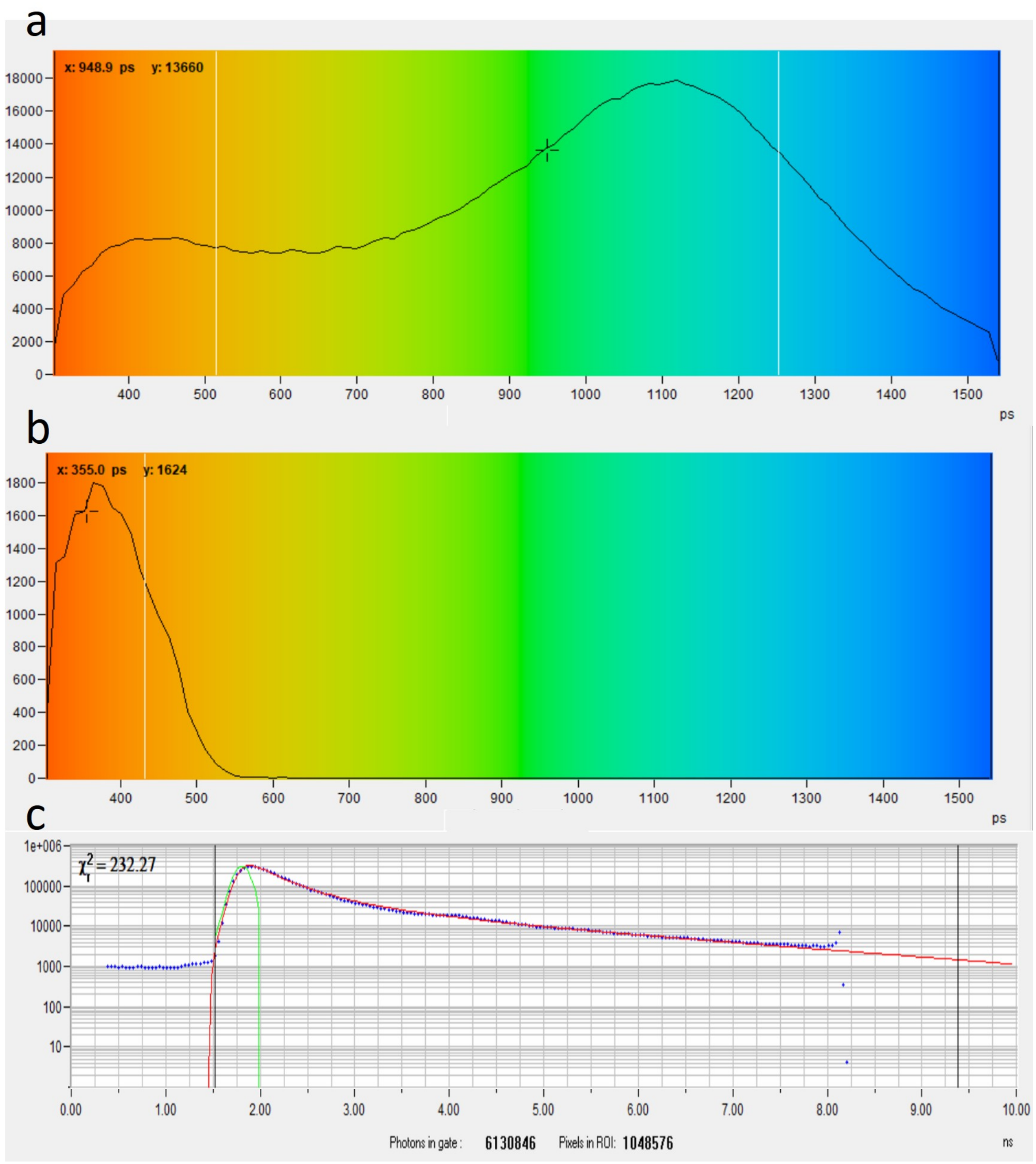

Fig 8 (a) a lifetime distribution over the whole image (b) the histogram of lifetime of the segmented region (lipofsucion) (c) the decay curve window of SPCImage displays the sum up decay curve for all pixels in the segmented area in a single decay curve, the blue dots are the photon numbers in the subsequent time channel, the green curve is the IRF, and the red curve is a fit with the model function.
